# Feasibility and Preliminary Effectiveness of the ChulaCancer Mobile Chatbot for Supportive Care of Patients With Breast or Colorectal Cancer Receiving Chemotherapy: Pilot Randomized Controlled Trial

**DOI:** 10.2196/86149

**Published:** 2026-06-01

**Authors:** Narawitch Sompornpailin, Thiti Susiriwatananont, Virote Sriuranpong, Suebpong Tanasanvimon, Chanida Vinayanuwattikun, Piyada Sitthideatphaiboon, Nattaya Poovorawan, Nattaya Teeyapun, Nicha Zungsontiporn, Nussara Pakvisal, Poonnakarn Panjasriprakarn, Bussaba Trakarnsanga, Napa Parinyanitikul

**Affiliations:** 1 Division of Medical Oncology, Department of Medicine Faculty of Medicine Chulalongkorn University & The King Chulalongkorn Memorial Hospital Bangkok Thailand; 2 Division of Neurology, Department of Medicine Faculty of Medicine Chulalongkorn University & The King Chulalongkorn Memorial Hospital Bangkok Thailand; 3 Pharmacy Department King Chulalongkorn Memorial Hospital Bangkok Thailand

**Keywords:** chatbot, mobile health, chemotherapy, symptom management, quality of life, randomized controlled trial

## Abstract

**Background:**

Chemotherapy-related toxicities often lead to unscheduled health care use and diminished quality of life. Digital health interventions, such as chatbots, offer a scalable solution for supportive care; however, evidence regarding their effectiveness in resource-limited, low- and middle-income settings remains limited.

**Objective:**

This study aimed to evaluate the feasibility, use, and preliminary effectiveness of a closed-loop chatbot (ChulaCancer Chatbot) in reducing unscheduled hospital visits and stabilizing quality of life among patients receiving chemotherapy for breast or colorectal cancer.

**Methods:**

This pilot randomized controlled trial enrolled 40 patients at a single academic center in Thailand, randomized 1:1 to either ChulaCancer chatbot plus usual care or usual care alone. The primary end point was the proportion of unscheduled hospital visits due to chemotherapy-related toxicities within 12 weeks of treatment initiation. Secondary end points included longitudinal quality of life changes (30-item EORTC Quality of Life Questionnaire) measured at baseline, following chemotherapy cycle 2, and following cycle 4. Use metrics were extracted from the chatbot platform. Data were analyzed using the Fisher exact test and linear mixed-effects models.

**Results:**

The platform recorded 2393 total messages with a 70.5% (503/713) successful response rate for user-initiated queries. Unscheduled hospital visits occurred in 15% (3/20) of the chatbot group compared to 35% (7/20) of the usual care group (*P*=.24). While infection-related visits were similar between groups, the usual care group recorded multiple visits for low-acuity symptoms (eg, anxiety, headache, and edema) that were absent in the chatbot group. Regarding quality of life, the chatbot group demonstrated a significant mitigation of cancer-related fatigue following cycle 4 compared with the usual care group (*P*=.02 between groups). Additionally, the chatbot group significantly improved in global health status (*P*=.04) and avoided the decline in physical functioning observed in the control arm (*P*=.04).

**Conclusions:**

The integration of a closed-loop chatbot into oncology care is feasible and provides a potential secure triage mechanism that may reduce acute care use for low-acuity concerns. Future large-scale trials incorporating agentic artificial intelligence are warranted to further validate clinical and economic benefits.

**Trial Registration:**

Thai Clinical Trials Registry TCTR20251220014; https://tinyurl.com/5b6k3e63

## Introduction

Despite significant advances in systemic cancer therapies, chemotherapy remains a cornerstone of curative-intent treatment for numerous malignancies. A population-based modeling study estimated that the global demand for chemotherapy will increase by 53% by 2040, with lung, breast, and colorectal cancer representing the leading indications. This projected increase is expected to place an unprecedented strain on the global oncology workforce and existing supportive care resources [1].

Chemotherapy is associated with a broad range of adverse events, including nausea, vomiting, diarrhea, mucositis, fatigue, and peripheral neuropathy. While severe toxicities require immediate clinical intervention, many symptoms are mild to moderate and can be safely managed at home through timely education, symptom monitoring, and appropriate supportive care [2,3]. However, the high volume of information provided at treatment initiation is often overwhelming for patients, making it difficult to apply general instructions to their specific, real-time symptoms [4,5]. This disconnect may lead to delayed care for serious toxicities or, conversely, avoidable hospital visits for manageable symptoms.

Mobile health interventions offer a scalable solution to these gaps by providing accessible, individualized support outside the clinical setting. Supported by the steady growth of smartphone penetration and mobile internet access in Thailand [6], chatbot platforms provide an increasingly viable channel for delivering on-demand, symptom-specific guidance and reinforcing self-management protocols. Preliminary evidence suggests that chatbot-assisted monitoring is feasible and may reduce chemotherapy-related adverse events and unscheduled hospitalizations [7-9]. Nevertheless, robust evidence comparing the effectiveness of chatbot-supported care against standard-of-care remains limited.

This study aimed to evaluate the feasibility, acceptability, and preliminary effectiveness of the ChulaCancer Chatbot—a Thai-language digital assistant—for the supportive management of chemotherapy-related adverse events in patients with early-stage breast and colorectal cancer.

## Methods

### Study Design and Setting

This prospective, single-center, open-label, randomized controlled trial (RCT) evaluated the feasibility and preliminary effectiveness of the ChulaCancer Chatbot—a proactive and interactive supportive care platform—when integrated into the standard of care. This study was conducted and reported in accordance with the CONSORT (Consolidated Standards of Reporting Trials) guidelines (Multimedia Appendix 1). The study was conducted at the outpatient chemotherapy unit of King Chulalongkorn Memorial Hospital in Bangkok, Thailand. In the intervention arm, the chatbot was used as a digital adjunct to existing institutional supportive care protocols, whereas the control arm received standard care alone.

### Ethical Considerations

The study protocol was approved by the institutional review board of the Faculty of Medicine, Chulalongkorn University (institutional review board number 0366/67), and written informed consent was obtained from all participants prior to enrollment. Participants received no financial or nonfinancial compensation for their participation in this study. Data collection, storage, and processing were conducted in strict accordance with Thailand’s Personal Data Protection Act. The chatbot explicitly identified itself as an automated assistant and included a clear medical disclaimer stating it did not replace professional clinical judgment. All user data were deidentified prior to content analysis, with personal identifiers removed to maintain participant anonymity.

### Chatbot Development and Usability Testing

The ChulaCancer chatbot was developed using the BOTNOI platform (BOTNOI Co Ltd) and integrated into the LINE messaging application (LINE Corporation), the most prevalent messaging platform in Thailand [10]. The chatbot’s full interactive functionality is accessible only via smartphone and tablet interfaces. The platform facilitated the design of the user interface, automated dialogue flows, natural language processing for intent recognition, and user interaction management.

**Figure 1:**
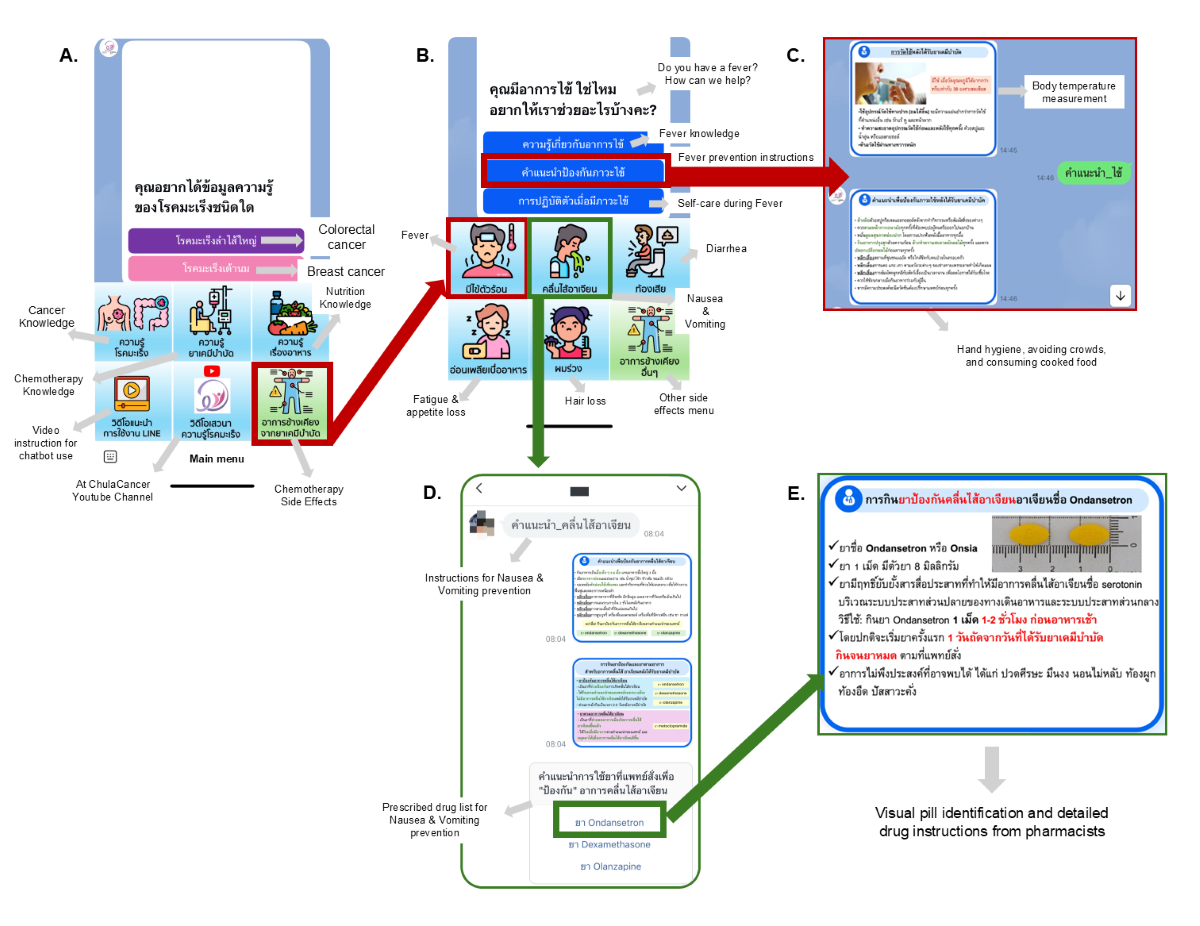
Operational architecture and user interface of the ChulaCancer chatbot: (A) main navigation menu for cancer-specific modules, (B) symptom-based interface featuring icon-based navigation, (C) self-care protocols for fever, (D) interactive decision support for nausea and vomiting prophylaxis, and (E) pharmacist-verified medication administration guides with visual pill identification.

The core content was adapted from existing institutional patient education materials that focused on five core modules: (1) general malignancy information, (2) chemotherapy regimen-specific education, (3) oncology nutrition, (4) supportive medication guidance, and (5) self-management for common toxicities (Figure 1). To ensure evidence-based accuracy, the system was commissioned following a multidisciplinary validation of the content by medical oncologists and oncology pharmacists. A closed-loop architecture was used to maintain clinical consistency and safety. Prior to the RCT, a formative usability study was conducted with 15 patients and 3 health care professionals. The system demonstrated high acceptability: 88.9% (16/18) of participants agreed or strongly agreed with the chatbot’s usability, 94.4% (17/18) reported overall satisfaction, and 100% (18/18) perceived it as clinically beneficial.

### Study Population and Randomization

Eligible participants included adults (aged >18 years) with early-stage breast or colorectal cancer scheduled for neoadjuvant or adjuvant chemotherapy. For breast cancer, eligible regimens included doxorubicin and cyclophosphamide or docetaxel and cyclophosphamide; for colorectal cancer, capecitabine and oxaliplatin was included. Participants required an Eastern Cooperative Oncology Group performance status of 0 to 2 and the ability to use the LINE application via a smartphone or tablet. Patients who could not personally navigate the application were eligible if a primary caregiver could facilitate the interaction. Participants were randomized in a 1:1 ratio to either the ChulaCancer chatbot group or the usual care group, stratified by cancer type. The usual care group received standard education, including a printed guidebook and links to educational videos.

### Intervention: ChulaCancer Chatbot

Participants randomized to the intervention arm were provided with access to the ChulaCancer LINE chatbot as a supplement to standard usual care. The proactive component involved the delivery of automated messages containing educational videos and evidence-based content regarding nutrition and the management of adverse events. These messages were timed to reach patients 1 week following chemotherapy administration during cycles 1 to 4. Simultaneously, the interactive component allowed participants to access the platform at their own discretion. Through a hierarchical navigation menu, users could engage in real-time, symptom-specific triage and receive immediate self-care recommendations for common chemotherapy-related toxicities. The platform remained accessible to either the patient or their primary caregiver throughout the study period and for 4 weeks following the final chemotherapy administration of the last enrolled participant.

### Outcomes and Statistical Analysis

The primary end point was the proportion of unscheduled hospital visits due to chemotherapy-related toxicities, assessed 12 weeks after the initiation of the first cycle. Secondary end points included quality of life, measured by the Thai version of the 30-item EORTC Quality of Life Questionnaire [11] at baseline, following chemotherapy cycle 2, and following chemotherapy cycle 4. Additionally, the incidence of grade >= 3 adverse events (Common Terminology Criteria for Adverse Events version 5.0) was assessed throughout the study duration. Chatbot use metrics were extracted via the BOTNOI platform. Categorical variables were compared using Fisher exact test. Longitudinal quality of life changes were analyzed using linear mixed-effects models. Statistical significance was set at *P*<.05. All analyses were performed using Stata (version 19; StataCorp).

## Results

### Participant Characteristics

**Figure 2:**
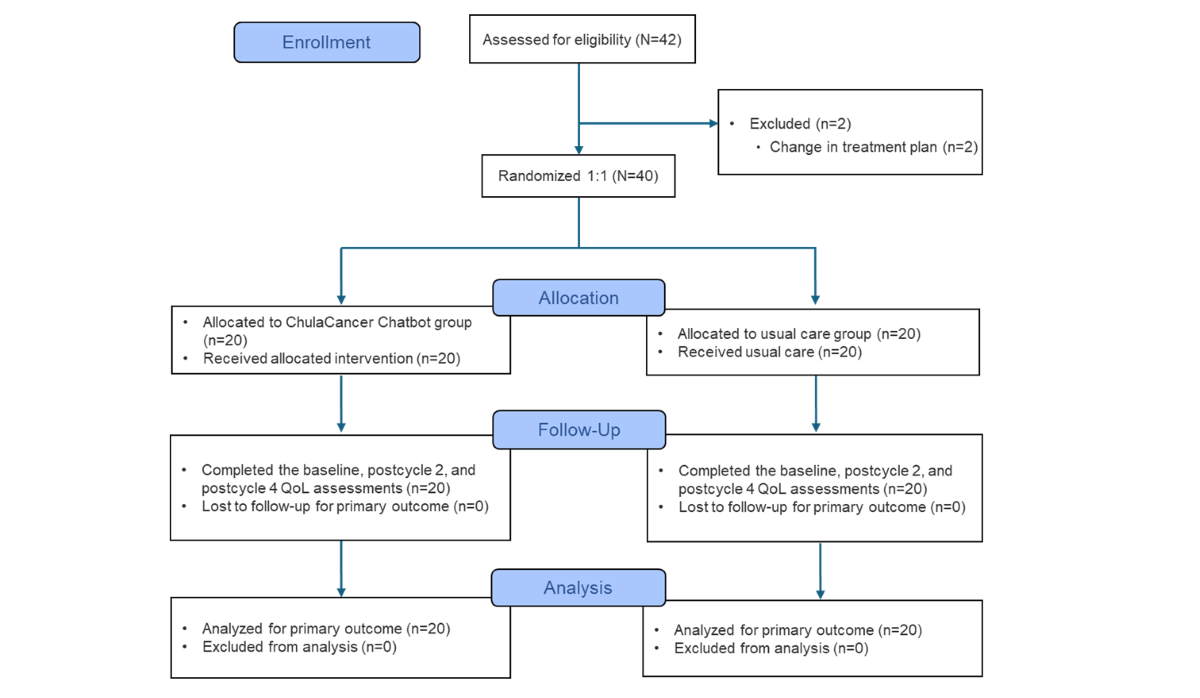
CONSORT (Consolidated Standards for the Reporting of Trials) flow diagram of the study. QoL: quality of life.

A total of 40 patients were enrolled between December 2024 and May 2025, with 20 participants randomized to each group (Figure 2). The median age of the overall cohort was 57 (IQR 47-64) years, and 80% (n=32) were female. Most participants exhibited good functional status, with 92.5% (n=37) having an Eastern Cooperative Oncology Group performance status of 0 or 1. Baseline demographic and clinical characteristics, including age, BMI, and education level, were well balanced between the chatbot and usual care groups (*P*>.05 for all comparisons). Detailed baseline characteristics are summarized in Table 1.

**Table 1:** Baseline characteristics of participants.

Characteristics			ChulaCancer chatbot (n=20)		Usual care (n=20)		*P* value	
Age (years), median (IQR)			57 (45-64)		57 (49-63)		.71	
Female, n (%)			17 (85)		15 (75)		.69	
BMI (kg/m^2^), mean (SD)			24.2 (4.7)		25.5 (6.4)		.47	
**Education, n (%)**								.56
	Primary school	5 (25)		4 (20)				
	Secondary school	1 (5)		3 (15)				
	Bachelor’s degree or higher	14 (70)		13 (65)				
Eastern Cooperative Oncology Group performance status of 1, n (%)			18 (90)		19 (95)		.99	
**Primary cancer, n (%)**								.99
	Breast	14 (70)		14 (70)				
	Colorectal	6 (30)		6 (30)				
**Chemotherapy setting, n (%)**								.72
	Adjuvant	14 (70)		16 (80)				
	Neoadjuvant	6 (30)		4 (20)				

^a^ECOG: Eastern Cooperative Oncology Group performance status.

### ChulaCancer Chatbot Use

During the operational period, the ChulaCancer chatbot recorded 22 unique new users, including the 20 randomized patients assigned to the intervention arm and 2 primary caregivers. The platform facilitated a total of 2393 messages, which included 713 incoming messages initiated by users. The chatbot successfully responded to 503 (70.5%) queries, while 210 (29.5%) messages were categorized as nonresponded. A total of 497 unique intents were triggered during the study. These were categorized into navigational menu selections (n=166) and content-specific queries (n=331). Analysis of the content-specific interactions revealed that participants primarily sought disease education and supportive care guidance. The most frequently accessed modules included cancer-related knowledge (61 intents), nutrition (27 intents), nausea and vomiting (26 intents), fever (12 intents), and diarrhea (11 intents).

### Unscheduled Hospital Visits Due to Chemotherapy-Related Toxicities

Unscheduled hospital visits related to chemotherapy toxicities occurred in 15% (3/20) patients in the chatbot group and 35% (7/20) patients in the usual care group (*P*=.24). In the chatbot group, visits were primarily due to infection (n=2) and diarrhea (n=1). In the usual care group, reasons for visits were more diverse, including infection (n=2), gastrointestinal symptoms (n=2), and other conditions such as leg edema, headache, and anxiety (n=3). The distribution of visit types is detailed in Table 2.

**Table 2:** Unscheduled hospital visits due to chemotherapy-related toxicities and clinical reasons.

Outcome		ChulaCancer chatbot (n=20), n (%)	Usual care (n=20), n (%)	*P* value
Unscheduled visit		3 (15)	7 (35)	.24
**Type of visit**				
	Outpatient clinic	1 (5)	3 (15)	—^a^
	Emergency department	1 (5)	3 (15)	—
	Hospitalizations	1 (5)	1 (5)	—
**Primary reason, n (%)**				
	Infection	2 (10)	2 (10)	—
	Gastrointestinal side effects	1 (5)	2 (10)	—
	Other	0 (0)	3 (15)	—

^a^Statistical significance was not calculated for subcategories due to the small number of events.

### Adverse Events

Most adverse events were mild (grade 1-2) across both cohorts. Grade >3= toxicities were infrequent, occurring in 5% (1/20; diarrhea) of the chatbot group and 10% (2/20; diarrhea and vomiting) of the usual care group, with no statistically significant difference between arms (*P*=.99).

### Quality of Life

**Figure 3:**
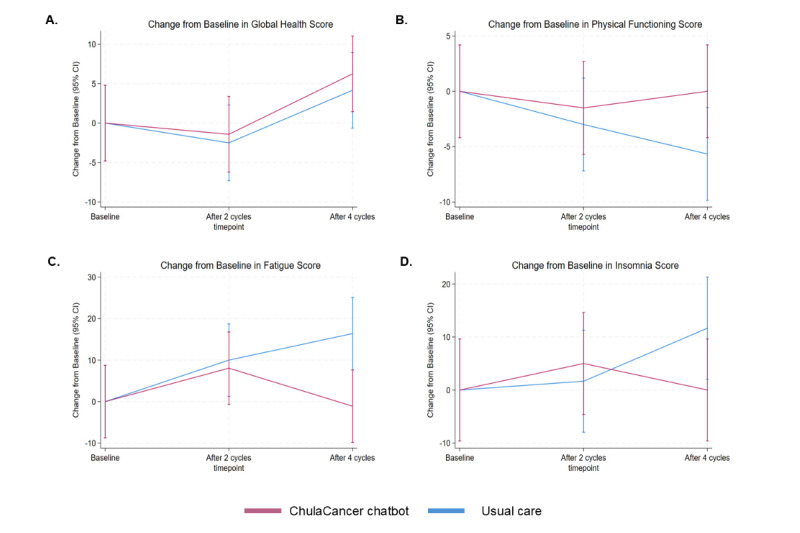
Longitudinal changes in quality of life (30-item EORTC Quality of Life Questionnaire) domains from baseline to after chemotherapy cycle 4: (A) global health status, (B) physical functioning, (C) fatigue, and (D) insomnia. Data are presented as mean change from baseline with 95% CIs. The chatbot group (pink) demonstrated a significant improvement in fatigue score compared to the usual care group (blue) at the end of cycle 4 (*P*=.02).

Quality of life assessments demonstrated significant variations between groups following the completion of chemotherapy cycle 4. In the global health status domain, the chatbot group showed significant improvement from baseline after cycle 4 (+6.25; *P*=.04), whereas no significant change was observed in the usual care group. Regarding physical functioning, the chatbot group maintained baseline levels after cycle 4 (0.00; *P*=.99), while the usual care group experienced a significant decline (−5.67; *P*=.04). The most notable difference was observed in fatigue scores. Following cycle 4, the usual care group reported a significant increase in fatigue (+16.4; *P*=.001), while the chatbot group remained stable (−1.11; *P*=.82), resulting in a significant between-group difference (*P*=.02). Insomnia scores followed a similar numerical trend with an increase in the usual care group after C4 (+11.7; *P*=.07) compared to no change in the chatbot group, although this was not statistically significant (*P*=.99; between-group *P*=.19). These findings are summarized in Table 3 and illustrated in Figure 3. Comprehensive data for all functional domains and symptom scales of the 30-item EORTC Quality of Life Questionnaire are provided in Multimedia Appendix 2.

**Table 3:** Longitudinal changes in quality of life (30-item EORTC Quality of Life Questionnaire) from baseline.

Domain or symptoms and group		Change after cycle 2 (95% CI)	*P* value^a^	Change after cycle 4 (95% CI)	*P* value^a^	Between-group *P* value^b^	
**Global health status**							.62
	Chatbot	−1.42 (−7.3 to 4.5)	.68	+6.25 (0.4 to 12.2)	.04		
	Usual care	−2.5 (−8.4 to 3.4)	.41	+4.2 (−1.7 to 10.1)	.17		
**Physical functioning**							.13
	Chatbot	−1.5 (−6.8 to 3.8)	.58	0.0 (−5.3 to 5.3)	.99		
	Usual care	−3.0 (−8.3 to 2.3)	.27	−5.7 (−10.9 to −0.4)	.04		
**Fatigue**							.02
	Chatbot	+8.1 (−2.1 to 18.2)	.12	−1.1 (−11.2 to 9.0)	.83		
	Usual care	+10.0 (−0.1 to 20.1)	.05	+16.4 (6.3 to 26.5)	.001		
**Insomnia**							.20
	Chatbot	+5.0 (−7.6 to 17.6)	.44	0.0 (−12.6 to 12.6)	.99		
	Usual care	+1.7 (−10.9 to 14.2)	.80	+11.7 (−0.9 to 24.2)	.07		

^a^Within-group change from baseline.

^b^Between-group difference after cycle 4.

## Discussion

### Principal Findings

This pilot RCT demonstrates that integrating a chatbot into the standard usual care workflow for patients receiving chemotherapy is feasible and provides encouraging signals for reducing unscheduled hospital visits and improving quality of life. These results align with previous reports suggesting that digital health interventions are promising tools for supportive care in oncology [12,13]. While the reduction in unscheduled hospital visits did not reach statistical significance, the observed trend in the reduction of outpatient and emergency department use is clinically noteworthy for a small pilot cohort.

A key observation was the absence of unscheduled visits for other clinical reasons—such as anxiety, headache, or leg edema—in the chatbot group, whereas these accounted for 43% (3/7) unscheduled visits in the usual care group. This suggests that the platform may provide sufficient triage and psychological reassurance, effectively addressing patient concerns that might otherwise lead to unnecessary hospital visits. This is further supported by the numerical improvements and stabilization seen across several quality of life domains, including global health status, physical functioning, and insomnia.

The most significant finding of this study was the chatbot’s ability to mitigate fatigue, a multidimensional and distressing symptom influenced by physical, emotional, and cognitive factors. The significant between-group difference observed after cycle 4 chemotherapy suggests that the chatbot group was better able to manage cumulative toxicity compared to the usual care group, who experienced a spike in fatigue levels. While the chatbot group showed less impact on acute physical symptoms such as nausea and diarrhea, its consistent stabilizing effect on broader functional domains suggests that timely, proactive communication and self-management are effective for general supportive care.

The chatbot’s closed-loop design was a deliberate choice to ensure medical accuracy and patient safety by restricting responses to predefined, peer-reviewed content. While this architecture successfully automated 70.5% of responses, its inherent lack of flexibility for out-of-scope inputs resulted in a 29.5% nonresponse rate. With the rapid advancement of large language models, the transition to agentic artificial intelligence (AI) represents a logical next step in overcoming these limitations. Agentic AI could provide more comprehensive and clinically appropriate medical advice while maintaining the necessary guardrails for oncology care—a field currently undergoing active investigation [14,15].

Despite these encouraging findings, several limitations inherent to the pilot nature of this study must be acknowledged. The small sample size and relatively short follow-up period may have limited the statistical power to detect differences in secondary clinical outcomes. Furthermore, the cohort consisted primarily of patients with early-stage disease and high functional status, which may limit the generalizability of these results to more advanced oncology populations. However, these constraints provided a controlled environment to establish the safety and feasibility of digital intervention.

### Conclusions

These findings provide preliminary evidence supporting the integration of conversational agents into the care of patients receiving chemotherapy, particularly in resource-limited settings where oncology resources are often strained. This pilot study demonstrates that a chatbot intervention is a feasible supplement to standard oncology care, offering potential benefits in stabilizing quality of life and reducing high-acuity health care use. While the reduction in unscheduled hospital visits did not reach statistical significance in this small cohort, the observed trends and the significant mitigation of cancer-related fatigue indicate promising clinical utility. Future research with larger, more diverse populations is necessary to validate these clinical outcomes and assess the long-term cost-effectiveness of digital supportive care. Furthermore, exploring the transition from closed-loop systems to more flexible agentic AI models may address current technical limitations and enhance the delivery of comprehensive, clinically appropriate support in oncology.

## Appendix

Multimedia Appendix 1 CONSORT-eHEALTH checklist (V 1.6.1).

Multimedia Appendix 2 Longitudinal changes in 30-item EORTC Quality of Life Questionnaire domains and symptom scales from baseline (n=40).

## Abbreviations

AI: artificial intelligence
RCT: randomized controlled trial

## Data Availability

The datasets generated and analyzed during this study are included in this published paper and its supplementary information files.
