# Supplementary material for: Feasibility and Preliminary Effectiveness of the ChulaCancer Mobile Chatbot for Supportive Care of Patients With Breast or Colorectal Cancer Receiving Chemotherapy: Pilot Randomized Controlled Trial
Source: JMIR Form Res. 2026 Jun 1;10:e86149. doi: 10.2196/86149 (PMC13270165; doi:10.2196/86149)
Supplement: Multimedia Appendix 2 [file formative_v10i1e86149_app2.docx]

**Supplementary material**

**Supplementary Table 1.** Longitudinal changes in EORTC QLQ-C30 domains and symptom scales from baseline (N = 40).

| Domain / Symptom | Timepoint | Group | Mean Change | 95% CI | P-value  (within-group change from baseline) | P-value  (between group) |
| --- | --- | --- | --- | --- | --- | --- |
| Global Health Status | After C2 | Usual Care | -2.5 | [−8.40,3.40] | 0.406 | 0.799 |
|  |  | Chatbot | -1.42 | [−7.31,4.48] | 0.682 |  |
|  | After C4 | Usual Care | +4.17 | [−1.73,10.06] | 0.166 | 0.625 |
|  |  | Chatbot | +6.25 | [0.35,12.15] | 0.038 |  |
| Functional Scales |  |  |  |  |  |  |
| Physical Functioning | After C2 | Usual Care | -3 | [−8.27,2.28] | 0.265 | 0.693 |
|  |  | Chatbot | -1.5 | [−6.77,3.78] | 0.578 |  |
|  | After C4 | Usual Care | -5.67 | [−10.94,−0.39] | 0.035 | 0.137 |
|  |  | Chatbot | 0 | [−5.27,5.28] | 1 |  |
| Role Functioning | After C2 | Usual Care | -6.67 | [−13.96,0.63] | 0.073 | 0.752 |
|  |  | Chatbot | -5 | [−12.29,2.29] | 0.179 |  |
|  | After C4 | Usual Care | -5.83 | [−13.12,1.46] | 0.117 | 0.526 |
|  |  | Chatbot | -2.5 | [−9.79,4.79] | 0.501 |  |
| Emotional Functioning | After C2 | Usual Care | +0.42 | [−7.09,7.92] | 0.913 | 0.608 |
|  |  | Chatbot | +3.20 | [−4.31,10.70] | 0.404 |  |
|  | After C4 | Usual Care | -0.42 | [−7.92,7.09] | 0.913 | 0.555 |
|  |  | Chatbot | +2.78 | [−4.73,10.28] | 0.468 |  |
| Cognitive Functioning | After C2 | Usual Care | +7.50 | [−0.91,15.91] | 0.08 | 0.114 |
|  |  | Chatbot | -2.08 | [−10.49,6.33] | 0.627 |  |
|  | After C4 | Usual Care | +2.08 | [−6.33,10.49] | 0.627 | 0.583 |
|  |  | Chatbot | +5.41 | [−2.99,13.82] | 0.207 |  |
| Social Functioning | After C2 | Usual Care | +0.83 | [−10.51,12.18] | 0.886 | 0.919 |
|  |  | Chatbot | +1.67 | [−9.68,13.01] | 0.773 |  |
|  | After C4 | Usual Care | +8.33 | [−3.01,19.68] | 0.15 | 0.497 |
|  |  | Chatbot | +2.78 | [−8.57,14.12] | 0.631 |  |
| Symptom Scales |  |  |  |  |  |  |
| Fatigue | After C2 | Usual Care | +10.00 | [−0.11,20.11] | 0.053 | 0.79 |
|  |  | Chatbot | +8.06 | [−2.06,18.17] | 0.118 |  |
|  | After C4 | Usual Care | +16.39 | [6.28,26.50] | 0.001 | 0.016 |
|  |  | Chatbot | -1.11 | [−11.22,9.00] | 0.829 |  |
| Nausea and Vomiting | After C2 | Usual Care | +4.16 | [−3.41,11.74] | 0.281 | 0.277 |
|  |  | Chatbot | +10.11 | [2.53,17.68] | 0.009 |  |
|  | After C4 | Usual Care | +10.83 | [3.26,18.41] | 0.005 | 0.661 |
|  |  | Chatbot | +8.44 | [0.87,16.01] | 0.029 |  |
| Pain | After C2 | Usual Care | +1.67 | [−6.62,9.95] | 0.693 | 0.676 |
|  |  | Chatbot | +4.17 | [−4.12,12.45] | 0.324 |  |
|  | After C4 | Usual Care | +10.83 | [2.55,19.12] | 0.01 | 0.486 |
|  |  | Chatbot | +6.67 | [−1.62,14.95] | 0.115 |  |
| Dyspnea | After C2 | Usual Care | +1.67 | [−7.15,10.49] | 0.711 | 0.239 |
|  |  | Chatbot | +9.17 | [0.35,17.99] | 0.042 |  |
|  | After C4 | Usual Care | +5.83 | [−2.99,14.65] | 0.195 | 0.793 |
|  |  | Chatbot | +4.17 | [−4.65,12.99] | 0.355 |  |
| Insomnia | After C2 | Usual Care | +1.67 | [−10.90,14.23] | 0.795 | 0.713 |
|  |  | Chatbot | +5.00 | [−7.57,17.57] | 0.435 |  |
|  | After C4 | Usual Care | +11.67 | [−0.90,24.23] | 0.069 | 0.198 |
|  |  | Chatbot | 0 | [−12.56,12.57] | 1 |  |
| Appetite Loss | After C2 | Usual Care | +11.67 | [−0.60,23.93] | 0.062 | 0.187 |
|  |  | Chatbot | +23.34 | [11.07,35.60] | <.001 |  |
|  | After C4 | Usual Care | +10.00 | [−2.27,22.26] | 0.11 | 0.572 |
|  |  | Chatbot | +15.00 | [2.74,27.27] | 0.017 |  |
| Constipation | After C2 | Usual Care | +15.00 | [3.65,26.35] | 0.01 | 0.684 |
|  |  | Chatbot | +18.33 | [6.98,29.68] | 0.002 |  |
|  | After C4 | Usual Care | +10.00 | [−1.35,21.35] | 0.084 | 0.839 |
|  |  | Chatbot | +11.66 | [0.32,23.01] | 0.044 |  |
| Diarrhea | After C2 | Usual Care | +3.33 | [−7.24,13.91] | 0.537 | 0.126 |
|  |  | Chatbot | +15.00 | [4.42,25.28] | 0.005 |  |
|  | After C4 | Usual Care | +10.00 | [−0.58,20.57] | 0.064 | 1 |
|  |  | Chatbot | +10.00 | [−0.57,20.58] | 0.064 |  |
| Financial Difficulties | After C2 | Usual Care | -15 | [−27.83,−2.16] | 0.022 | 0.15 |
|  |  | Chatbot | -1.66 | [−14.50,11.17] | 0.799 |  |
|  | After C4 | Usual Care | -1.66 | [−14.49,11.17] | 0.8 | 0.472 |
|  |  | Chatbot | -8.33 | [−21.16,4.51] | 0.203 |  |
